# Editorial for the Special Issue on Soft Robotics: Design, Fabrication, Modeling, Control and Applications

**DOI:** 10.3390/mi14010027

**Published:** 2022-12-23

**Authors:** Thanh Nho Do

**Affiliations:** 1 Graduate School of Biomedical Engineering, Faculty of Engineering, UNSW Sydney, Kensington Campus, Sydney, NSW 2052, Australia; tn.do@unsw.edu.au; 2 Tyree Institute of Health Engineering, UNSW Sydney, Sydney, NSW 2052, Australia

Living environments often require high adaptation from biological organisms, such as altering their shape and mechanical properties. In contrast to rigid counterparts, soft materials are associated with many advantages, such as low cost and high compliance. In recent years, the creation of functional devices from soft condensed matter or soft robotics has received increasing interest and is expected to transform how humans safely interact with intelligent machines. We anticipate that soft robotics will play a vital role in developing innovative devices for use in medicine, biomimetics, haptics, and industries.

In this Special Issue on “Soft Robotics: Design, Fabrication, Modeling, Control, and Applications”, we focus on the latest advancement of several emerging soft robotic technologies, ranging from soft robotic grippers and flexible manipulators to jumping, legged robots, as well as the advanced modeling and control of soft bodies. Both fundamentals and applications are also covered in this issue. Out of the eight articles published in this Special Issue, seven are original research papers, and one is a review paper. Three papers were submitted from China; three were from Egypt; one was from collaborative research between China and Japan; one collaborative work was from South America, including Peru, Brazil, and Venezuela; and one was from Russia.

In particular, Chen et al. [1] developed a new modeling method for soft robotic arms. The authors combined the constant curvature model with Euler–Bernoulli beam theory to quickly estimate soft arm deformation under the presence of an external force. Youssef et al. [2] proposed a data-driven approach using machine learning to model the kinematics of Soft Pneumatic Actuators (SPAs) where an Echo State Network (ESN) architecture was utilized to predict the SPA’s tip position in three axes without requiring any precise model. Chen et al. [3] fabricated a fabric-reinforced soft manipulator driven by a water hydraulic system and developed a dynamic model of both the soft manipulator and hydraulic system. One of the novelties of this approach was the use of an improved Newton–Euler iterative method, which comprehensively considered the influence of inertial force, elastic force, damping force, and combined bending and torsion moments of the soft arm. EI-Agroudy and colleagues [4] presented a new framework to develop a soft robotic body, including details on the design, fabrication, finite element modeling, and experimental validation of a soft pneumatic robotic finger, enabling a fast approach to achieve the desired soft body. Broyko et al. [5] introduced a multi-physics simulator that could simulate the most significant physical processes in ionic polymer–metal composites (IPMC). Kang et al. [6] introduced a bio-inspired take-off mechanism based on the coordination of upper and lower limbs, which could improve the jumping height of a quadruped robot with a manipulator. Daza et al. [7] proposed an autonomous navigation approach for mobile robots in indoor environments based on the principles of proxemic theory, integrated with classical navigation algorithms. Finally, Youssef and colleagues [8] provided a comprehensive review of recent developments in the soft robotics field, with a focus on the underwater application frontier.

We hope this Special Issue will solicit work from experts in the field aimed at solving the existing challenges of soft robotics to develop “softer and smarter” robotic systems.
